# Suicidality in the Light of Schema Therapy Constructs, i.e., Early Maladaptive Schema and Schema Modes: A Longitudinal Study

**DOI:** 10.3390/jcm12216755

**Published:** 2023-10-25

**Authors:** Anna Grażka, Klara Królewiak, Klaudia Sójta, Dominik Strzelecki

**Affiliations:** 1 Department of Affective and Psychotic Disorders, Medical University of Lodz, 92-216 Lodz, Poland; annagrazka.psycholog@gmail.com (A.G.); klaudia.krakus@stud.umed.lodz.pl (K.S.); 2 Faculty of Psychology, SWPS University, 03-815 Warszawa, Poland; kkrolewiak@swps.edu.pl

**Keywords:** early maladaptive schema (EMS), schema mode, suicide, psychiatric disorder, dimensional approach, longitudinal study

## Abstract

(1) Background: The search for new strategies to diagnose people at risk of suicide and to help them is highly significant in view of the still high rate of suicidality. Schema therapy and its core constructs, i.e., early maladaptive schemas (EMSs) and schema modes, correspond to both directions. (2) Methods: This study compared the severity of EMSs and schema modes in a clinical group of suicide risk, a clinical non-suicidal group, and a control group. Intragroup comparisons were also conducted between times of crisis and psychological stability. The evaluation was supported by controlling for the psychopathological symptoms presented, following the dimensional concept. (3) Results: The unquestionable relevance of the disconnection/rejection domain in suicidality has been proven. The importance of EMSs from other domains, especially during psychiatric crises, was confirmed. Among the schema modes, child and Punitive Parent modes proved to be the most significant. There were changes in coping modes but of a lesser effect size. The protective importance of the Healthy Adult and Happy Child modes was also proven. (4) Conclusions: The results provide an indication for practitioners about the EMSs and schema modes most associated with suicide risk. They can also serve as a framework for deepening the issue of identifying and preventing suicidality in schema therapy.

## 1. Introduction

### 1.1. Suicidality and Theoretical Approaches

Each year, an estimated 703,000 people worldwide die by suicide, based on data from the World Health Organization (WHO). In 2019, 1.3% of deaths were the result of suicide. It is more than the percentage of deaths from malaria, HIV/AIDS, breast cancer, war, or homicide. The global mean is about nine suicide deaths per 100,000 people, with significant differences between countries (from 2 to 80 per 100,000) [1].

Although the WHO’s global statistics over the period 2000–2019 note a decline in the overall number of suicides by about 36%, the phenomenon is nevertheless of such significance that a reduction in mortality from this cause has been recognized as one of the overarching global goals and included in the United Nations Sustainable Development Goals. Such a statement also appears within the WHO’s Mental Health Action Plan 2013–2020 (extended to 2030). It postulates a one-third reduction in suicide deaths [1]. Many pre- and postvention efforts are being taken, but there is still a need for work in this area.

The theoretical approach to suicide has changed over the years. Initially (dating back 3000 years), it was treated as prohibited behavior, and committing it was considered a sin. Exceptions were made when self-inflicted death was seen as a means to avoid dishonor or escape a life of infirmity or irreversible misery [2]. This approach evolved in the 19th century when papers began describing suicide as an illness and the person in a suicidal crisis as needing care. Moreover, attention began to focus on the concept of a state that predisposes to suicide and to protective factors [3]. Most contemporary models regard a wide range of determinants of suicidal behavior. The most commonly considered are biological, psychological, and social variables [4]. Several current theoretical approaches to suicide are presented below to justify considering the issue in the context of schema therapy constructs and the significance of examining them at different time points of crisis.

One trend in understanding suicide is theories treating it as an escape. Baumeister, who represents this view in his work, described six steps leading to suicide [5]. First, there is a discrepancy between the excessively high standards the individual wants to achieve and the perceived reality. Second, the individual attributes failure as an immanent feature of himself, which leads to the emergence of guilt. Third, the distorted self-awareness is subjected to further comparisons with the unmet standards. Fourth, it triggers a wave of painful emotions. Fifth, the individual attempts to escape into a state of numbness, focusing on specific sensations and immediate goals. Ultimately, this results in diminished behavioral control and leads to self-destructive behavior, including suicide [5].

A theory that recognizes suicide as an escape is also Shneidman’s Theory of Psychache [6]. In it, the author defined ten determinants of suicide: search for a solution; suspension of consciousness; unbearable psychological pain; unmet psychological needs; lack of hope; helplessness; ambivalence toward life and death; limitation of viable alternatives; escape from life itself; communication of intention to take one’s own life; and dysfunctional coping patterns. As a core construct, he identified unbearable psychological pain, from which the individual seeks respite, finding it only in death. Schneidman distinguished between two types of needs that should be satisfied: primary, i.e., biological, and secondary—psychological. The second category includes love, belonging, a sense of control, a positive self-image, and meaningful relationships. Failure to satisfy them constitutes the development of the aforementioned psychological pain [6].

Linehan, who worked with patients diagnosed with borderline personality disorders, had similar observations. The researcher pointed out the relevance of suicidality in perceiving life as unbearable [7]. She recognized that suicide risk consists of many factors, from life crises, environmental stressors, and problematic relationships with others to difficulties in professional life and health problems, all of which—in the way of dysfunctional coping strategies (including emotional dysregulation)—give birth to stress, reduce the chance of improving the quality of life, and take away hope.

The significance of a sense of hopelessness for suicidality was assessed in their study by Beck et al. [8], proving that people presenting high levels of this variable are 11 times more likely to commit suicide. Thus, it was noted that it is a better predictor of suicide risk than the level of depression.

The mentioned theories point out the multiple predictors co-occurring simultaneously, identifying key variables somewhat differently. They do not clearly distinguish between thoughts, tendencies, and readiness to act. Such a distinction has begun to be made within the so-called second-generation models, which treat suicide as a process in which we observe a transition from ideation to action [9]. These models indicate that the current psychiatric disorder diagnosis may be one of many triggers, but the key is the configuration of numerous motivational and volitional variables that vary at subsequent stages of the process leading to suicide.

An example is Rudd’s cognitive-behavioral six-stage model [10]. The first phase includes predisposing factors, such as a mental or personality disorder or childhood trauma. In the second phase, we find external and internal stressors. The third phase deals with suicidal thoughts and feelings of hopelessness. In addition, the identified fourth and fifth phases include access to means of committing suicide and describing the individual’s emotional state (such as anger). According to the author, a suicide attempt, treated throughout the model as phase six, may already occur after phase three.

The processuality is also pointed out by Joiner et al. [11] in Interpersonal–Psychological Theory of Suicidal Behavior (IPT), assuming that suicide is committed by individuals who “want and can” do it. In his/her view, an individual at suicide risk is characterized by a high level of severity of two constructs, i.e., (I) perceived burdensomeness, that is, feeling of being a burden on the family, friends, or society, and (II) thwarted belongingness, which is the experience of being separated from other people [11]. According to Joiner, an additional prerequisite is the ability to commit suicide, which has to do with fearlessness, which stems from a dysfunction of the amygdala acquired through experiences that involve experiencing pain.

Another second-generation model, namely, the Three-Step Theory (3ST) created by Klonsky and May [12], assumes that suicide (I) results from a combination of pain and hopelessness, (II) is contrary to one’s sense of connection to others, and (III) requires moving from thoughts to attempts through acquired factors such as fearlessness in the face of death or increased tolerance of physical pain, all of which contribute to the ability to commit suicide.

A much more comprehensive description of suicide is found in the Integrated Motivational and Volitional Model of Suicidal Behavior (IMV) by O’Connor et al. [13]. In it, the researchers emphasize the importance of a three-stage process preceding the suicidal act. The premotivational phase includes the conditions that constitute the background (i.e., the individual’s biological, genetic, and cognitive vulnerability, with social and environmental context) and the triggering events. The motivational phase begins with the onset of feelings of failure and humiliation, which leads to a sense of entrapment and, finally, to the emergence of suicidal thoughts. The volitional phase involves a transition from suicidal thoughts to action with the help of components, i.e., acquired ability, but also environmental, social, psychological, or physiological variables [13].

The most recent concept discussed is the two-phase stress–diathesis model of suicidal behavior presented by Mann et al. [14], considering the results of numerous studies regarding the involvement of genetic and epigenetic factors in suicidal behavior. They proved that increased suicide risk is associated with higher activity of the hypothalamic–pituitary–adrenal axis. Suicide victims were found to have lower levels of brain-derived neurotrophic factor (BDNF). Elevated levels of peripheral inflammatory biomarkers were also confirmed. These and several other biological variables constitute vulnerability. Meanwhile, stressors can be a combination of external factors (e.g., demanding life events) and internal factors (e.g., current mental disorders) [13].

Recent years have abounded in the search for biological factors in the context of suicide risk; however, the contribution of variables previously studied is sustained in the reports presented. Díaz-Oliván et al. [4], based on a systematic review of theoretical models, attempted to create a unified theoretical model of suicidal behavior. This model is mainly derived from the IMV by O’Connor et al. [13]. It emphasized the processuality of the discussed phenomenon, described biological variables in more detail, and expanded the categories of exemplary moderators. Risk factors specific to the child and adolescent population are also included. The authors point out the need to look at the linearity of the phenomenon. Classically, suicidal thoughts lead to plans, which are transformed into action. However, it turns out that it is possible to make a suicide attempt based only on plans without the mediation of suicidal thoughts [4]. This issue requires further research.

Simultaneous with establishing predictors in some studies, researchers focus on determining the strength of the association of individual factors with suicide risk. According to the results obtained in a meta-analysis by Favril et al. [15], based on autopsy studies, the strongest associations are in the clinical domain. The presence of any mental disorder was associated with a more than 10-fold increase in the risk of committing suicide. More than two-thirds of those who committed suicide reported psychiatric disorders at the time of death. At highest risk were those with a diagnosis of depressive disorder. Also confirmed was the importance of a personality disorder diagnosis (especially borderline personality disorder). As many as 29% of the cases studied had at least one suicide attempt in the past [15].

Besides determining predictive factors and their strength, studies conducted to identify protective factors against suicide are also noteworthy. In recent years, there has been a growing focus on these issues. For comparison, typing in the keywords “protective factor” and “suicide” into the PubMed database results in the display of 860 records for the years 2019–2020, with almost as many papers (i.e., 857) reported in the ten years preceding this period (i.e., 2010–2018). Some researchers, such as Cheng et al. [16], treat protective factors as the inverse of risk factors (e.g., support [11,13] vs. no support). Others, including Appleby et al. [17], point to coexistence between them. In their opinion, protective factors are specific circumstances that act preventively in case of significant risk without changing the risk factors themselves.

Most focus in the literature has been given to the protective role of the family and social support network (including friends, school environment, or workplace). Cheng et al. [16], for example, proved that the prevention of early death by suicide (especially among adolescents) is favored by factors present in families, such as interest, attention, assistance in decision-making, acceptance, demonstrated support and bestowal of care to the exclusion of excessive control, shared activities, and a positive pattern of coping with daily struggles, with emotions, and in communication situations. Protective variables against suicide risk also identify self-esteem level, self-concept, problem-solving skills, and ability to use support [18].

### 1.2. Schema Therapy Constructs (EMSs and Schema Modes) and Their Links to Suicidality

The theories and research findings cited above directly relate to the concepts underlying schema therapy by Young et al. [19]. The author assumed that the optimal human development and satisfaction with life are determined by the adequate fulfillment of early childhood emotional needs, which are secure attachment, autonomy, a sense of competence and identity, freedom to express needs and emotions, spontaneity and play, and the need for realistic boundaries and self-control. Failure to meet these needs results in early maladaptive schema (EMSs) formations. According to Young, EMSs can be understood as filters that determine how we anticipate, order, and interpret the world around us. EMSs develop under the influence of negative life experiences and factors such as parenting styles or an individual’s temperament. A brief description of the 18 EMSs identified by Young et al. [19] is provided in Table 1.

Besides ESMs, schema modes are a core construct of Young’s theory. In contrast to ESMs, which are treated as trait beliefs, schema modes are viewed as a combination of a dominant emotional state, schema, and coping response occurring in an individual at a specific time [19,20]. A description of the 14 distinguished schema modes is presented in Table 2 [21].

Looking at the distinguished ESMs and schema modes and the mechanisms of their emergence, their involvement in the suicide crisis seems undeniable. Several theories (inter alia [4,6,10,11,12,13,16]) have raised the topic of the importance of the lack of satisfaction of early childhood emotional needs in the path of, for example, the occurrence of traumas, conditioning greater tolerance to pain and less fearlessness in the face of death. Many authors made references to difficult emotions such as helplessness [4,6,11,13], a sense of hopelessness [4,6,10,11,12,13,22], guilt [4,5,11,13], perceived burdensomeness [4,11,13], thwarted belongingness [4,11,12,13], internalized sense of failure and humiliation [5,11,13], or experiencing unbearable psychological pain and being trapped in it [4,6,11,13,23], which predominate in the experience of many EMSs (primarily from the disconnection/rejection and impaired autonomy/performance domains) and form some modes (especially the Vulnerable Child mode).

Dysfunctional coping strategies are also significant for suicidality, according to the theoretical assumptions cited [4,5,11,13,23]. In the schema concept, they can be classified into three groups, i.e., overcompensation (e.g., trying to meet excessive standards), avoidance (e.g., substance abuse), and subjugation (e.g., submissiveness in relationships). Considering their cognitive, emotional, physiological, and memory-related load, they form the so-called conditional EMSs (i.e., unrelenting standards/hypercriticality, approval seeking/recognition seeking, emotional inhibition, self-sacrifice, and subjugation), i.e., the ones that, according to Young, are used to cope with more primary, so-called unconditional schemas [19]. In the behavioral area, these strategies are expressed as non-adaptive coping modes (i.e., Compliant Surrender, Detached Protector, Detached Self-Soother, or Self-Aggrandizer).

Also noteworthy is considering mental disorders as a predictor of suicide risk [4,10,11,13,14]. It is known that among people with psychiatric disorders, there is a higher intensity of EMSs and dysfunctional schema modes and a lower severity of healthy schema modes compared to people without disorders [24,25,26,27,28,29,30,31,32]. It is further evidence that we should observe higher severity of EMS and dysfunctional coping modes and lower severity of adaptive modes in people with suicide risk.

There are several studies that have tested the association of EMS with suicide risk, most of them conducted in groups of patients suffering from specific psychiatric disorders [24]. The results indicate the most significant importance of EMS from the disconnection/rejection domain, which also persists when controlling for depressive, anxiety, or manic symptoms [25,26,27,28,29]. The strongest associations were recognized for the social isolation/alienation and defectiveness/shame schema. Impaired autonomy/performance was another domain in which there were links between EMSs and suicidality. In this domain, the most significant results were obtained for the dependence/incompetence and vulnerability to harm and illness patterns. The other domains showed associations with significantly smaller effect sizes.

To date, only a study by Leppännen et al. [30] has examined schema modes in patients diagnosed with borderline personality disorder, exhibiting parasuicidal and non-parasuicidal behaviors. Statistically significant higher scores were obtained in the suicidal group for the severity of four modes, namely, the Vulnerable Child, Angry Child, Detached Protector, and the Compliant Surrender.

### 1.3. Objectives of the Present Study

Available studies have dealt with ESMs and schema modes, comparing groups of patients with suicide risk (variously defined) with patients of the same diagnosis without suicide risk or, alternatively, with a healthy control group. Guided by the increasingly popular dimensional approach in psychiatric diagnosis [31,32], the present study compared EMSs and schema modes between three groups: (I) psychiatric-treated patients in suicidal crisis; (II) psychiatric-treated non-suicidal patients; and (III) a control group with no history of psychiatric treatment and no suicidal tendencies. The groups were assigned regardless of the nosological diagnosis, controlling the severity of psychopathological symptoms. Consistent with the results cited above, the highest severity of EMSs (especially in the disconnection/rejection domain) and dysfunctional schema modes were expected in the clinical suicide group and the lowest in the control group. An opposite relationship was predicted for the Healthy Adult and Happy Child modes (the lowest severity was expected in Group I, the highest in Group III).

Both constructs (EMS and schema modes) were also assessed in a repeated-measures model: in crisis and during a period of stabilization of the mental state. It was hypothesized that significant differences should occur in the severity of schema modes due to their variability over time, primarily in both clinical groups (suicidal and non-suicidal). It was assumed that changes could also apply to EMS, despite Young’s theoretical assumption of their constancy over time [19]. This hypothesis was justified by the fact that the questionnaire assessment of the construct in a crisis situation was likely to be distorted due to the subject’s emotional state. It would prevent him/her from fully identifying the EMS he/she would have access to in a situation of stabilization of his/her mental state [32]. It was expected that these patterns (regarding both schema modes and EMSs) would not apply to the healthy control group due to their greater stability in individuals without psychiatric disorders [19,33,34].

## 2. Methods and Design

### 2.1. Participants

This study included 126 adults with a range of ages from 18 to 65, who were classified into three groups: (I) currently psychiatrically treated with a current suicidal crisis (N = 39); (II) currently psychiatrically treated with no history of suicidal crisis (N = 36); (III) never psychiatrically treated with no history of suicidal crisis (control group, N = 51). Exclusion criteria included individuals with intellectual disability, with a diagnosis of organic disorders, partially or totally incapacitated, with somatic comorbidities in an unstable phase and chronic diseases of significant severity that could strongly affect mental status, and persons who did not meet the inclusion criteria and those who withdrew their consent to participate in this study during the course. The demographic characteristics of the participants divided into groups are shown in Table 3.

The control group was not statistically different regarding gender, age, education level, place of residence, and employment from the clinical non-suicidal group. The only difference was in marital status. However, there were slight statistically significant differences between the control group and the clinical suicide group for the above variables, with the exception of place of residence.

In the clinical group (i.e., groups I and II combined), 53 subjects (70.7%) declared a diagnosis of one of the following mental disorders in the course of their lives: depression; anxiety disorder; bipolar disorder; eating disorder; schizophrenia; or alcohol dependence. Forty participants (53.3%) declared the presence of a personality disorder, of which 26 (35.7%) had both types of disorders co-occurring. As the participants were recruited for this study at the diagnostic stage of their treatment, it was decided that the severity of individual psychiatric symptoms would be controlled using the SCL-90 scale. Such a solution was supported in some cases by the low degree of precision of classical criterion diagnosis (e.g., the diagnosis of mixed personality disorder or anxiety–depressive disorder) and its limited informative value in the context of the severity of other, often equally burdensome symptoms for the patient, such as somatization or anger levels. It also made it possible to look at the dynamics of symptom severity over time. The approach applied is in congruence with the dimensional model proposed as an alternative to the criterion approach in the Diagnostic and Statistical Manual of Mental Disorders (DSM-5; [31]).

According to additionally obtained data, 85.3% of patients in the clinical group had a history of at least one psychiatric hospitalization. All of the subjects in group I were currently hospitalized, while group II consisted of 66.7% currently hospitalized patients. The rest of the patients were undergoing outpatient treatment. A total of 45.3% of the subjects in the clinical group said they were staying under the regular care of a psychiatrist. A total of 18.7% attended irregular visits, while the rest denied outpatient psychiatric treatment. Three-quarters of this group were taking psychiatric medications (including antidepressants, anti-anxiety medications, and mood stabilizers) regularly, and 69.3% confirmed the use of psychotherapy. One in four respondents declared that they had some kind of chronic somatic disease in a stable phase.

Participants in this study were recruited over a period of about 10 months (from October 2022 to June 2023) within the departments of the Central Clinical Hospital in Lodz and a private psychotherapy practice run by the researchers. The timing of this study was determined by the availability of subjects, who were recruited successively from those who met the inclusion criteria. A control group was assembled by screening people from the researchers’ surroundings.

### 2.2. Materials

At the inclusion stage, we used questions from the Columbia Suicide Severity Rating Scale (C-SSRS; [35]) on suicidal thoughts and behaviors. The presence of (I) desire to die, (II) active suicidal thoughts, (III) consideration of methods, (IV) actual intent, (V) presence of a specific plan, and (VI) actual occurrence of behavior aimed at taking one’s own life were assessed.

Next, demographics and the presence of previous psychiatric and psychological treatment were collected. The main test battery consisted of the questionnaires described below.

- Young Schema Questionnaire—S3 (YSQ-S3)

To measure EMSs, this study used a short version of the schema questionnaire (YSQ-S3, [36]) in a Polish adaptation by Oettingen et al. (YSQ-S3-PL, [37]). The test items were acquired directly from the authors. The questionnaire consists of 90 statements to assess 18 early maladaptive schemas. There are five items for each factor. Respondents are asked to respond to each statement on a six-point Likert scale, where 1 means “completely untrue about me”, and 6 means “this describes me perfectly”. The results for each of the highlighted factors range from 5 to 30. The arithmetic mean is taken in comparisons. The higher the score, the higher the severity of the specific schema.

A validation study of the Polish adaptation of the questionnaire showed acceptable internal reliability (Cronbach’s α ranging from 0.62 to 0.81 for individual scales; [37]). Convergent validity testing confirmed positive mean correlations with scales measuring psychiatric symptoms. At the same time, negative correlations were obtained with self-efficacy and optimism, proving good divergent validity. In the current study, the reliability of individual scales was questionable to excellence, ranging from 0.6 to 0.94 (with a mean of 0.83) on the first measurement (crisis) and from 0.66 to 0.95 (with a mean of 0.83) on the second measurement (stability);

- Schema Mode Inventory (SMI)

To measure the schema modes, we used a Short SMI by Lobbesteal et al. [21] in a Polish adaptation by Grażka et al. [24]. The questionnaire was obtained directly from the authors. It consists of 112 items assigned to 14 factors. Each factor contains between 4 and 10 items. The factors are grouped into four domains (i.e., dysfunctional child modes, coping modes, parenting modes, and healthy modes, including Healthy Adult and Happy Child modes). The inventory is preceded by an instruction, according to which the respondent has to rate how much a given statement describes him on a six-point Likert scale, where 1 means “never or almost never”, and 6 means “all the time”. The results obtained are the arithmetic mean for the factor. The higher the score, the higher the severity of the factor. No cut-off point is provided.

The Polish adaptation is characterized by acceptable to excellent internal reliability indices (McDonald’s omega from 0.74 to 0.95). Satisfactory theoretical validity of subscales in both convergent and divergent aspects (using temperament and character dimensions, scales measuring anger in terms of trait and state, self-esteem, and coping strategies) was confirmed. In this study, internal consistency indices ranged from 0.67 to 0.95 (with a mean of 0.84) for the crisis measure and from 0.75 to 0.92 (with a mean of 0.84) for the stability measure, so it was taking values from questionable to excellent;

- Symptom Checklist (SCL-90)

Monitoring of the mental state of the subjects for the severity of various types of psychopathological symptoms was carried out using the SCL-90 by Derogatis et al., in Polish translation by Jankowski [38]. The scale allows for the measurement of nine types of symptoms, i.e., somatization, obsessive-compulsive, interpersonal severity, depression, anxiety, anger and hostility, phobia, paranoid ideation, and psychoticism. It consists of 90 statements, which the respondent rates on a five-point Likert scale, where 0 means “not at all” and 4 means “very strongly”. The higher the subscale score, the higher the severity of the symptom under study.

Admittedly, there is a Polish adaptation of a shortened version of the same questionnaire (SCL-27; [39]) with known psychometric properties, but the authors decided—with awareness of the limitations of such a procedure—to use the 90-item version, in view of the more comprehensive catalog of psychopathological symptoms. Thanks to this, we also controlled the level of psychotic, paranoid, and anger disorders, whose scales are not included in the shortened version. It is worth mentioning the fairly widespread use of the SCL-90 questionnaire, especially among patients with alcohol dependence syndrome, where the results obtained show fairly good facade validity. In the current sample, Cronbach’s α reliability coefficients were from good to excellent, ranging from 0.83 to 0.94 (with a mean of 0.88) in crisis and from 0.81 to 0.91 (with a mean of 0.87) in stability.

### 2.3. Procedure

Before the beginning of this study, permission was obtained from the Bioethics Committee of the Medical University of Lodz. Patients were included in this study shortly after their admission to the hospital (mostly on the second–third day after admission) or at the initial stage of psychotherapy (on the second–third meeting). The researchers, based on the available psychiatric or psychological examination, recorded the circumstances of initiation of current treatment (current suicidal crisis/lack of suicidal crisis) and information regarding the presence/absence of a suicidal crisis in the patient’s history. Participants in the control group were recruited from the surroundings of the researchers. They applied in response to an announcement sent by email and verbal communication. The group included people who declared that they had not experienced a suicidal crisis in the past and had never received psychiatric treatment. We tried to match the participants on gender and age, and we ultimately succeeded only for subgroup II.

The data obtained were collected face-to-face using a paper-and-pencil method. The control group participated in this study via an online survey. Each willing participant was introduced to the identity of the researcher and written information about the purpose and design of this study and was asked to sign an informed consent form. Participants were assured of the scientific nature of this study, the voluntary character of participation, the ability to withdraw consent at any stage of this study, and the complete anonymity of the data collected. Taking part in this study did not affect the medical and psychological care received.

Each subject was tested individually. First, an interview was conducted using the C-SSRS. Respondents who declared the presence of suicidal thoughts and tendencies were assigned to the first study group (clinical, suicidal). The others (declaring no presence of suicidal thoughts and tendencies over the course of their lives) formed the second study group (clinical). Subsequently, the subjects received a battery of three questionnaires to fill out (i.e., the YSQ-S3-PL, SMI, and SCL-90) preceded by a survey with demographic data and previous psychiatric/psychological treatment. In each group, at the first measurement, it was emphasized to go back in memory to the last crisis or difficult, unpleasant situation (referring to the associated thoughts, emotions, physiological symptoms, and the behavior undertaken) and evaluate the given statements from this perspective. The completion of the battery took approximately 45 min.

After four weeks (relatively after the suicide risk had ceased), the psychologist–researcher contacted the participants a second time to repeat the entire procedure. They were then asked to fill out questionnaires from the perspective of their current psychological state. To preserve the anonymity of the data, a letter code assigned to each patient was used to combine their results from two measurements. Every participant in this study interested in receiving his or her psychological characteristics could participate in a follow-up interview with the psychologist–researcher or provide an email address to which the description of the results was sent.

### 2.4. Statistical Analyses

To conduct statistical analyses, we used the ANOVA test with repeated measures to assess differences between means in EMS-s severity and schema modes in the distinguished three groups and the Student’s *t*-test within contrasts to assess the presence of detailed differences between groups. We decided that simply identifying differences between groups was insufficient, so we also assessed effect sizes, using η_p_^2^ and Perason’s r (after Field, [40]) to find out which of the study variables most co-varied with suicide risk. For the statistical calculations, the statistical package PS IMAGO PRO 8.0 and Statistica 13.3 were used.

## 3. Results

### 3.1. Characteristics of Participants Considering Severity of Psychopathological Symptoms

**Figure 1:**
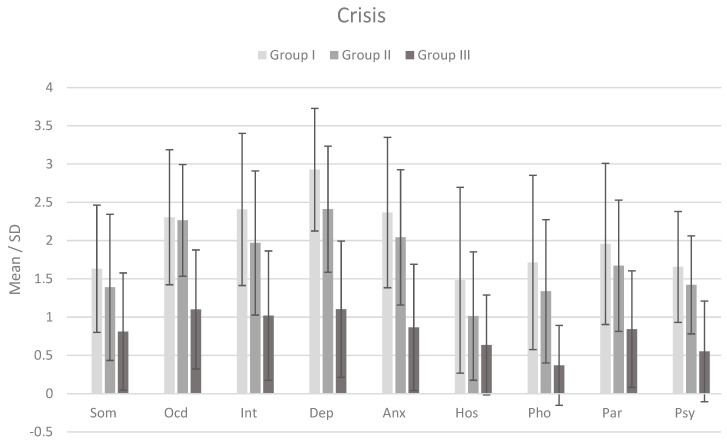
Mean and standard deviation (SD) of psychiatric symptom severity in each group (N = 126) during the crisis. Note: Som = Somatization; Ocd = Obsessive-compulsive; Int = Interpersonal severity; Dep = Depression; Anx = Anxiety; Hos = Anger and hostility; Pho = Phobia; Par = Paranoid ideation; Psy = Psychoticism.

Figure 1 shows the means and standard deviations for the severity of each symptom on the SCL-90 in the three groups during the crisis (first measurement). Using one-way analysis of variance (ANOVA) and post hoc analysis, it was shown that significant differences (at *p* ≤ 0.05) existed between each of the clinical groups (I and II) and the control group (III), while differences between clinical groups were non-significant (except for the depression dimension, where all differences were significant).

**Figure 2:**
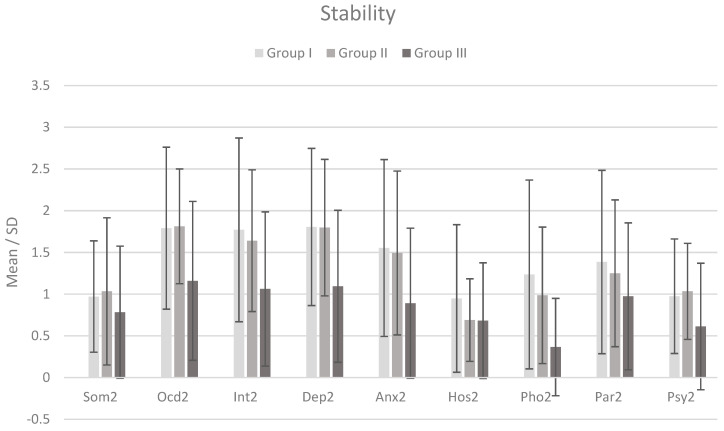
Mean and standard deviation (SD) of psychiatric symptom severity in each group (N = 94) during stability. Note: Som2 = Somatization; Ocd2 = Obsessive-compulsive; Int2 = Interpersonal severity; Dep2 = Depression; Anx2 = Anxiety; Hos2 = Anger and hostility; Pho2 = Phobia; Par2 = Paranoid ideation; Psy2 = Psychoticism.

For the second measurement (in stability), the results were distributed in a different way. Figure 2 shows the means and standard deviations within symptoms in each of the distinguished groups. Analyzing the differences between the means, it was found that the clinical groups did not differ in all measured symptoms. Only some of the differences between the clinical and control groups remained compared to the first measurement. The control group differed significantly from the clinical groups in obsessive–compulsive, depressive, and phobic symptoms. There were also differences between the control group and the clinical suicide group in the case of the interpersonal severity scale and anxiety scale. The other differences were not significant.

For cases where repeated measurements were obtained, the significance of differences between symptom severity during crisis versus stability in the distinguished groups was tested. It was proven that during the second measurement, the severity of all examined symptoms was significantly lower compared to the first measurement in both clinical groups (suicidal and non-suicidal). There were no statistically significant differences in the severity of the tested symptoms in the control group, with the exception of the paranoid ideation subscale, where there was an increase in the severity of this trait (see Table 4).

### 3.2. Intra- and Intergroup Differences between EMSs and Schema Modes—General Assessment

Table 5 shows the interaction effects between the distinguished groups considering the measurement time and their sizes (η_p_^2^). For the modes in which we obtained a non-significant two-factor effect, we also separately checked for the existence of between-subject (I, II, III) and within-subject (crisis vs. stability) differences.

It was proven that statistically significant differences (considering both effects) occur within nine EMSs, i.e., abandonment/instability, mistrust/abuse, defectiveness/shame, social isolation/alienation, dependence/incompetence, vulnerability to harm and illness, self-sacrifice, emotional inhibition, and punitiveness. The remaining EMSs showed significant inter- and intragroup differences examined separately for emotional deprivation, failure, insufficient self-control/self-discipline, subjugation, and negativity/pessimism; only between-subject significant differences were shown for enmeshment/undeveloped self, and only within-subject significant differences for approval seeking/recognition seeking and unrelenting standards/hypercriticality. In the case of only one ESM (i.e., entitlement/grandiosity) between the distinguished groups, no statistically significant differences were obtained. It is worth noting that at least medium (η_p_^2^ ≥ 0.06) to large effects (η_p_^2^ ≥ 0.14) were obtained with significant differences.

The same analysis was performed for schema modes. Table 6 demonstrates the interaction effects obtained for the distinguished groups over time and their sizes. For modes where the two-factor effect was found to be non-significant, a single-factor test of the effect was performed, obtaining within- and between-subject effects.

Considering the two-factor effect, statistically significant differences were obtained in the case of eight modes (i.e., Vulnerable Child, Angry Child, Impulsive Child, Compliant Surrender, Detached Protector, Punitive Parent, Healthy Adult, and Happy Child). When analyzing differences among the other modes, we found significant within- and between-subject differences (tested separately) in the case of the Enraged Child and Bully and Attack modes, and significant differences only in within-subjects in the case of the Undisciplined Child, Detached Self-Soother, and Demanding Parent modes. Self-Aggrandizer mode was the one that did not show either within- or between-subject differences. For significant differences, we observed at least medium (η_p_^2^ ≥ 0.06) to large (η_p_^2^ ≥ 0.14) effect sizes.

### 3.3. Intergroup Differences in EMSs and Schema Modes—Detailed Assessment

To interpret the effect above, we used contrast analysis. At the first stage, a detailed analysis of intergroup differences was conducted, comparing the clinical suicidal group (I) with the clinical non-suicidal group (II) and the clinical suicidal group (I) with the control group (III) (separately for crisis and stability). To evaluate the effect sizes as recommended by Field [40], we used Pearson’s r coefficient calculated according to the following formula: r_contrast_ = $\sqrt{\frac{t^{2}}{t^{2}+df}}$. In interpreting the r coefficient, we followed Cohen’s guidelines [41], according to which r = 0.10 means a small effect; r = 0.30 means a medium effect, and r = 0.50 means a large effect. Table 7 shows the results obtained in terms of EMSs.

As a preliminary description of the results, it should be noted that the highest severity of EMSs was observed in the suicidal group, while the lowest was observed in the control group. Comparing the clinical suicidal group with the clinical non-suicidal group in crisis, we obtained five statistically significant differences, including four from the disconnection/rejection area. The highest effect size occurred in the abandonment/instability schema (r = 0.425). For the remaining significant differences, effect sizes were within the medium range. When comparing the same groups in stability, a statistically significant difference was proven only for the self-sacrifice schema, with a small effect size.

Comparing the suicidal and the control group in crisis showed statistically significant differences in the 15 EMSs. Large effect sizes were obtained for four EMSs (from the disconnection/rejection domain). Eight EMSs had medium effect sizes, and the remaining had two small effect sizes. No differences only appeared for the schema of entitlement/grandiosity, approval seeking/recognition seeking, and unrelenting standards/hypercriticality. Comparing these groups in stability resulted in 11 statistically significant results. Regarding effect sizes, schemas from the disconnection/rejection domain once again dominated. Six medium effect sizes were obtained. The others were at a small level.

Intergroup comparisons according to the same formula were also carried out for the intensity of particular schema modes, as shown in Table 8.

Remarkably, the highest intensity of dysfunctional schema modes (i.e., child, coping, and parental) was observed in the suicidal group, while the lowest was in the control group. On the other hand, regarding healthy modes, the opposite trend was proven: the suicide group was characterized by the lowest intensity of these modes compared to the clinical non-suicidal and control groups, where the healthy modes tended to be the strongest.

There were seven statistically significant results when comparing the suicidal group with the clinical non-suicidal group in crisis, mainly within the child modes. Small effect sizes were obtained, with the highest observed for the Vulnerable Child mode (r = 0.355). Comparing the aforementioned groups in stability, statistically significant differences were obtained only for the Angry Child mode (with a small effect size).

When we compared the suicidal group with the control group in crisis, statistically significant differences were noted in the case of 12 schema modes (10 dysfunctional and two healthy). Large effect sizes were obtained for Vulnerable Child, Detached Protector, Punitive Parent, and two healthy modes (i.e., Healthy Adult and Happy Child modes). Medium effects were observed for five modes, and small effects were observed for the remaining modes. Comparing these groups in stability resulted in eight statistically significant differences with smaller effect sizes than the above. Six medium effect sizes (the largest for the Healthy Adult mode) and two small ones were obtained.

### 3.4. Differences in ESMs and Schema Modes between Crisis and Stability Times—A Detailed Assessment

At the next stage of contrast analysis, the intensity of each EMS and schema mode was compared comprehensively, considering the repeated measurement. Table 9 provides comparisons of the mean intensities of EMSs in crisis and stability (separately for each group) and the effect sizes achieved.

It should be noted that there was a decrease in the severity of the EMS under consideration for all statistically significant results. This means that, contrary to the assumption of constancy of EMSs, there is a group of individuals whose EMSs intensity changes under the influence of a crisis situation.

The results of the suicide group (I) were analyzed, and statistically significant differences were obtained in the case of 14 EMSs: four from the disconnection/rejection domain; four from the impaired autonomy/performance domain; three from the other-directedness domain; and three from the hypervigilance/inhibition domain. There were non-significant differences for four EMSs: emotional deprivation; entitlement/grandiosity; approval seeking/recognition seeking; and unrelenting standards/hypercriticality. The largest effect sizes were obtained for emotional inhibition (r = 0.544) and defectiveness/shame (r = 0.529). In the case of the other EMSs, effect sizes for significant differences were medium (except for enmeshment/undeveloped self and self-sacrifice, where a small effect size was obtained).

Comparing the mean severity of EMS in crisis and during the period of stability in the clinical non-suicidal group (II) yielded only four statistically significant results. There were differences in the following EMS: approval seeking/recognition seeking; emotional inhibition; unrelenting standards/hypercriticality; and punitiveness. Effect sizes were medium or small.

In the control group (III), none of the differences between the means in crisis and stability proved significant. This fact supports the lack of impact of the crisis on the severity of individual ESMs in the non-psychiatric treatment group.

As a final step, it was verified whether there were statistically significant differences between crisis and stability (separately for each group) across schema modes. The results, including an assessment of effect sizes, are shown in Table 10.

Again, all statistically significant differences proven within dysfunctional modes involved a decrease in severity, while differences within healthy modes involved an increase in severity. Considering the suicide group (I), it was shown that statistically significant differences existed between almost all schema modes (with the exception of the Self-Aggrandizer mode), comparing the time of crisis and stability. Large effect sizes were obtained for Vulnerable Child mode (r = 0.654), Punitive Parent mode (r = 606), Detached Protector mode (r = 0.559), Happy Child mode (r = 0.506), and Compliant Surrender mode (r = 0.501). For the remaining modes, effect sizes were at the medium level, except for three modes (i.e., Detached Self-Soother, Bully and Attack, and Demanding Parent mode), where they were small.

In the non-suicidal clinical group (II), it was noted that statistically significant differences between the crisis and stability states were found for ten schema modes, with smaller effect sizes compared to those observed in the suicidal group. Differences with a small and medium effect size occurred in the child modes (except for the Angry Child mode), two coping modes (i.e., Compliant Surrender and Detached Protector) with a small effect, the Punitive and Demanding Parent modes (with a medium and small effect, respectively), and the Healthy Adult (with a medium effect) and Happy Child (with a small effect).

Within the control group (III), a statistically significant difference between crisis and stability was shown for only one mode, i.e., Detached Self-Soother (with a small effect size).

## 4. Discussion

### 4.1. Main Findings

The objective of the present study was to investigate whether there were differences between the psychiatric-treated suicide risk group, the psychiatric-treated non-suicidal group, and the control group by severity of EMSs and schema modes, comparing the crisis and stabilization of the psychiatric condition. It is the first study of this type on suicidality to simultaneously compare the two core constructs of schema therapy on intra- and intergroup dimensions.

The analysis was preceded by assessing the severity of psychopathological symptoms observed in each group. Since the two clinical groups were mostly similar in this aspect and differed from the control group, we can conclude that the suicide crisis was a significant differentiating factor between them. Moreover, we noted a significantly higher severity of psychopathological symptoms among subjects participating in the repeated measurement during the crisis compared to the stabilization period, confirming the validity of the self-report.

#### 4.1.1. The Combined Relevance of Schema Therapy Constructs to Suicidality

The overall analysis resulted in a number of significant two-factor interaction effects for both EMSs (especially in the disconnection/rejection domain) and schema modes (for child modes, in particular Vulnerable Child, two coping modes, i.e., Compliant Surrender and Detached Protector, for Punitive Parent mode and both healthy modes). For the remaining variables, the differences were less pronounced. The exceptions were the entitlement/grandiosity schema and the Self-Aggrandizer mode, where no differences were noted. These constructs relate to compensatory beliefs about one’s own superiority, uniqueness, or privilege. If they persist in a crisis, they do not lead to feelings of unbearable emotions, helplessness, or hopelessness, and thus, the individual does not feel the need to self-destruct.

A recurring pattern at the effect size assessment stage was that the suicidal clinical group had the highest severity of EMSs and dysfunctional schema modes and the lowest severity of healthy modes (i.e., Healthy Adult and Happy Child modes) compared to the non-suicidal clinical and control groups. In the control group, the results found were the opposite. All of the above findings were repeated when comparing crisis and stability, i.e., in crisis, we obtained higher intensities of EMSs and dysfunctional schema modes and lower intensities of healthy modes in each of the distinguished groups compared to times of stability.

The results of this study confirm the high significance of unpleasant emotional states described in the conceptions of many authors (i.e., [4,5,6,12,13,30]. Feelings such as a sense of hopelessness, helplessness, uncontrollable emotional pain, guilt, perceived burdensomeness, thwarted belongingness, or a sense of entrapment are strongly associated with the activation of EMSs from the disconnection/rejection domain and child modes, which are manifestations of suffering resulting from the inability to satisfy one’s own emotional needs. It is close to the views of authors who have pointed out the value of fulfilling these needs in triggering [4,6,10,11,12,13,16] but also preventing [16,18] a suicide crisis.

These feelings are reinforced by the voice of a heavy Punitive Parent mode, which was discussed in his concepts by Baumeister [5], noting the sense of failure attributed to the self in not achieving impossible goals. Jointer et al. [11] and O’Connor et al. [13] also emphasized the weight of critical voice for suicidality, pointing to beliefs about being a burden and not belonging to a group.

Lower contributions to the suicidal group in crisis were made by so-called conditional schemas [19] and coping strategies, which dooms the individual to experiencing painful ESMs and remaining in the intense emotions of child modes. This study also confirmed an under-representation of Healthy Adult and Happy Child modes in both clinical groups compared to the control group, which is consistent with the findings of Cheng et al. [16] or Pereira et al. [18] on protective factors. These authors highlight the importance of meeting basic early childhood needs, for which healthy modes are responsible.

However, it is worth remembering that in interpreting the results, we should also consider other explanations. The clinical groups were characterized by a number of psychopathological symptoms. While their intensity did not differ between the suicidal and clinical non-suicidal groups, comparison with the control group proved significant differences, which may have amplified the effect sizes. Additionally, the clinical groups mainly consisted of hospitalized patients. Admittedly, hospitalization was a consequence of the subjects’ psychiatric condition, but this fact could also have influenced the slightly different profile of EMSs and schema modes. It would be worth verifying the mentioned explanations of the obtained results in further studies.

#### 4.1.2. EMSs and Suicidality

Analyzing the results obtained in the context of EMSs in more detail, it is essential to note the highest importance of the disconnection/rejection domain for suicidality. Almost all of the EMSs from this domain (excluding emotional deprivation) and the self-sacrifice schema from the other-directedness domain measured in crisis occurred at significantly higher levels in the suicidal group compared to the clinical non-suicidal and control group. In stability, these differences were confirmed only in comparison with the control group (except for the self-sacrifice schema), which shows the very high significance of the study situation to the results obtained.

The results are consistent with the systematic review findings of studies linking suicidality to EMSs [42]. They indicate that the schemas from these areas and the self-sacrifice schema allow us to differentiate between people at risk of suicide and psychiatric inpatients without suicidal history, but only in a crisis situation, which is a new observation made in the present study.

Noteworthy were the outcomes obtained within the impaired autonomy/performance domain, which, while not indicating its differential role in suicidality, show its weight in the occurrence of clinical disorders in general. This issue would require further verification.

Concerning EMSs comparing crisis to stability time, a significant decrease in results was obtained mainly in the suicidal group for all schemas from the impaired autonomy/performance domain and almost all schemas from the other domains. Noteworthy are two patterns, i.e., the defectiveness/shame and emotional inhibition, in which we obtained the largest effect sizes. These results are congruent with the concept of Baumeister [5], who claims that a sense of failure to measure up to unachievable standards is crucial in the way leading to suicide. It is a source of painful emotions (defectiveness/shame schema), from which the individual tries to escape into a numbed state (emotional inhibition schema).

In addition, in the non-suicidal clinical group, it was proven that there was a significant decrease in severity for four EMSs (i.e., emotional inhibition, unrelenting standards/hypercriticality, punitiveness, and approval seeking/recognition seeking) between crisis and stability. It is easy to see that three of them (with the exception of punitiveness) are conditional schemas identified by Young. It leads to the conclusion that this group in crisis activated (admittedly dysfunctional) coping mechanisms. In the control group, ESMs in crisis did not differ from the time of stability, which is consistent with Young’s theoretical assumptions about the relative sustainability of these constructs over time [19].

#### 4.1.3. Schema Modes and Suicidality

Considering schema modes in relation to suicide in crisis, we proved that the child modes (i.e., Vulnerable Child, Angry Child, Impulsive Child, and Enraged Child mode), one of the parent modes (Punitive Parent mode), and two of the coping modes (Detached Protector and Compliant Surrender mode) were the strongest differentiators between the suicidal group and non-suicidal clinical group. Moreover, comparing the suicide group with the control group (both in crisis and stabilization), there was a significantly higher intensity of the remaining dysfunctional child modes and of both the healthy modes.

These results were confirmed when comparing groups in crisis and stability. It can be observed that the child mode and the Punitive Parent mode stood out with the greatest strength of differences. Also of great importance were two dysfunctional coping modes, the Detached Protector mode and the Compliant Surrender mode, consistent with concepts treating suicide as an escape [5,6,23].

In the non-suicidal clinical group, there were differences primarily in the child and Punitive Parent modes, with a lower than in the suicidal group effect size, which supports the feeling of intense emotions in crisis related to a critical approach to oneself. In the control group, the intensity of only one mode (i.e., Detached Self-Soother) decreased significantly. This result confirms the more constant modes in healthy subjects (regardless of the circumstances). Nevertheless, it should be remembered that in the control group, the respondents imagined a crisis situation (without participating in it at the time of this study), which may have distorted the results.

Another important observation was made regarding the intensity of healthy modes. Respondents in crisis declared lower severity of Healthy Adult and Happy Child modes in both clinical groups versus stability. In contrast, the control group reported no change in the intensity of these modes over time. There is evidence that participants in suicidal crises and non-suicidal participants with significant severity of psychopathological symptoms “turn off” healthy modes in favor of dysfunctional modes. Consequently, while they are able to use them for stability, this is severely hampered during the crisis. Several studies on the schema modes in psychiatric disorders confirm these results (e.g., [30,32]). In comparison, people in the control group have a relatively stable level of healthy modes, regardless of the presence of a crisis.

### 4.2. Practical Implications

The conclusions obtained may have broad clinical applications. They can be helpful in assessing suicide risk at the diagnostic stage. The diagnostician, seeing a profile of ESMs and schema modes dominated by schemas from the disconnection/rejection domain and the Vulnerable Child or Punitive Parent mode, should pay particular attention to further suicide risk investigation.

In some cases, the use of questionnaires (such as the YSQ-S3 or SMI), which do not explicitly ask about suicidal thoughts or tendencies, will be more revealing to the patient (because they are associated with lower levels of anxiety) and may lead to a more authentic description of the mental state. However, it should be emphasized that testing ESMs or schema modes is not a strategy that can replace a reliable suicide risk assessment.

Knowledge of the EMS and schema modes most associated with suicide can also guide psychotherapists–practitioners in assessing suicide risk. We would encourage extreme caution, especially with apparent changes in the profile over time, most notably an increase in schema modes from the disconnection/rejection domain, child and Punitive Parent modes, and decreasing access to healthy modes. Changes in the opposite direction to those discussed above achieved over time will also provide valuable information on the patient’s condition improvement and the reduction in suicide risk.

Moreover, in the schema therapy practice, special attention is paid to the need to activate the Sensitive Child mode. In controlled conditions, this is a highly desirable situation. Through the therapeutic techniques used (including limited repeated parenting), we have a possibility to modify the patient’s automatic dysfunctional ways of responding. Outside the practice, however, activating this mode involves the risk of the patient experiencing very intense emotions with limited access to healthy modes, which can turn into a suicidal crisis. That is why it is of value to develop healthy modes simultaneously so that patients acquire the skills for self-care while experiencing difficult emotions.

### 4.3. Limitations

Besides the valuable conclusions drawn from this study for both theory and practice, at the same time, it is necessary to mention its limitations. It is the first study of its kind, and, therefore, it does not entitle us to generalize the results obtained. We should treat its conclusions with particular caution.

The first issue is the weaknesses of the selection stage of the study group. The subgroups formed were not numerous enough and not fully matched regarding gender and age. Predominantly, they consisted of women and people between the ages of 25 and 35. In addition, the repeated measurement stage characterized a reasonably high dropout rate (especially in the control group). All of the above shortcomings may have led to distorted results. Because few studies are showing the effect of gender and age so far on EMSs severity (i.e., [43,44]) and no studies describing the distribution of schema modes, it would be of value to fill this gap in the future.

Similarly, there is a lack of information on the effect of seasonality on ESMs and schema modes, whereas the data in the present study were collected for about ten months. With knowledge of the impact of seasons, days of the week, or even time of day on suicidality (see, e.g., [45]), it would be meaningful to design a study that controlled for these variables, including in the context of schema therapy constructs.

Another questionable issue was the respondents’ self-report of experienced psychopathological symptoms. Admittedly, this study tried to move away from a categorical approach to a dimensional approach (therefore not focusing on specific diagnoses); nevertheless, it would have been worthwhile to additionally introduce an external screening questionnaire for assessing psychopathological symptoms, thereby reducing the influence of the subjective feelings of the participants.

Limitations certainly include the issue of an imagined crisis in the control group versus experiencing a real crisis (resulting in psychiatric treatment) in the other groups, which obviously could have undermined the reliability of the results. Weakened comparisons with the control group may also have resulted from the high number of dropouts during this study. Perhaps the feedback was not enough incentive to participate in this study in the case of this group. More effective motivators would need to be considered.

Arbitrarily, it was also decided to perform a second measurement of EMSs and schema modes about four weeks after the first measurement. In most cases, this allowed for an improvement in psychological state and especially the cessation of suicide risk. At the same time, however, the crisis termination does not necessarily equate to stability. These conditions would need to be better specified in subsequent studies.

### 4.4. Future Research

The limitations mentioned above prompt us to improve this study with better sample selection. It would be worthwhile to conduct it on a larger, more diverse group, controlling for variables such as the severity of specific psychopathological symptoms, age, and gender. Meanwhile, this study can become the basis for further, more detailed scientific analyses, looking not only at correlation patterns but also checking for causality.

In view of models considering the processuality of suicidality, it would be interesting to distinguish groups given different levels of suicide risk to compare them by severity of EMSs and schema modes, which was not possible in the present study due to too small a sample size. It seems that the first stages of the crisis should be associated with the activation of numerous EMSs (initially unconditional and then conditional), which would result in the behavioral dimension of increased use of dysfunctional schema modes and weakened access to healthy modes. However, this issue requires detailed empirical verification.

Also noteworthy are the preliminary findings regarding coping with crises among psychiatric healthy individuals. In this study, the control group was based on an imaginary crisis, which could lead to distortions. Therefore, it is postulated that similar longitudinal analyses are to be conducted in an experimental model or in an actual crisis situation (e.g., related to work or relationship difficulties) after the stressor has ceased. This issue would require further study.

Initially, this study also found a significant role for the impaired autonomy/performance domain as a differentiator between those with psychiatric disorders and those without disorders. It would be interesting to see if, for example, working in therapy on schemas from this domain correlates significantly with a decrease in the severity of psychopathological symptoms.

## 5. Conclusions

To the best of our knowledge, the present study is the first in which the issue of suicidality was examined in connection with EMSs and schema modes, considering the variability of these constructs over time. Analyses were made after checking the level of psychopathological symptoms in each distinguished group (not limited to criterion diagnosis), following the approach postulated in the latest classifications of mental disorders (DSM-5, ICD-11).

The results confirm the unquestionable significance of the disconnection/rejection domain in suicidality. They also point to the activation of EMSs from other domains, especially during a crisis of psychological distress.

Among the schema modes, child and Punitive Parent modes are the most crucial for suicide risk. Some changes are noted within the coping modes, nevertheless of a lesser size. This study also confirmed the protective importance of the Healthy Adult and Happy Child modes. The described regularities are found in people with the severity of psychopathological symptoms, leading them to seek psychiatric treatment. Changes of this type do not appear in people without psychiatric treatment who have not experienced a suicidal crisis.

In conclusion, the current study definitely broadens the knowledge of EMSs and schema modes in patients at risk for suicide. With the awareness of the above-mentioned limitations, its results can support both the diagnostic and therapeutic process, increasing the chance of identifying patients at high risk of suicide. In addition, the results obtained enrich the data on the scientific validation of the concept of schema therapy, laying the groundwork for further analysis.

## Figures and Tables

**Table 1:** Description of the 18 EMSs grouped into five domains.

Domain	Early Maladaptive Schema	Description
Disconnection/Rejection	Abandonment/instability	Beliefs about the instability and unreliability of available relationships.
	Mistrust/abuse	The expectation that others will intentionally hurt, abuse, humiliate, cheat, lie, manipulate, or gain an advantage.
	Emotional deprivation	A belief that the desire to have a usual level of emotional support from others will not be sufficiently fulfilled.
	Defectiveness/shame	A sense of incompleteness, of being inadequate, unwanted, or inferior, which makes it impossible to be loved and accepted.
	Social isolation/alienation	A feeling that a person is disconnected from the world, different from other people, and not part of society.
Impaired autonomy/performance	Dependence/incompetence	A general perception of being unable to cope with daily duties without having significant help from others.
	Vulnerability to harm and illness	Experiencing an excessive fear of the possibility of a catastrophe (health, emotional, external) that cannot be avoided.
	Enmeshment/undeveloped self	Over-involvement emotionally and closeness to at least one person, leading to impairment of complete individuation or normal social development.
	Failure	The individual’s belief that he has completely failed and is incompetent compared to others in fields of achievement.
Impaired limits	Entitlement/grandiosity	An individual’s conviction that he or she is superior to others and entitled to special rights and benefits.
	Insufficient self-control/self-discipline	The difficulties in developing adequate self-control and tolerance to frustration.
Other directedness	Subjugation	Perception of the need to surrender control of others’ needs and emotions to avoid feelings of anger, revenge, or being abandoned.
	Self-sacrifice	Focusing on voluntarily fulfilling the needs of others over one’s own.
	Approval seeking/recognition seeking	It is an overly excessive desire to gain appreciation, respect, or attention from others or to conform to them at the expense of developing a strong, authentic self.
Hyper vigilance/inhibition	Negativity/pessimism	A constant concentration on the negative aspects of life (e.g., pain, death, loss, conflict, possible mistakes), accompanied by overlooking or underestimating the positive aspects.
	Emotional inhibition	Excessive inhibition of spontaneous behavior, feelings, and communication with others to avoid disapproval, feelings of embarrassment, or losing control.
	Unrelenting standards/hypercriticalness	Feeling the need to meet high internalized behavioral and performance standards to avoid criticism.
	Punitiveness	The conviction that people should be heavily punished for their faults.

**Table 2:** Description of 14 schema modes included in four categories.

Category	Schema Modes	Description
Maladaptive Child Modes	Vulnerable Child	The individual experiences a sense of unhappiness, anxiety, sadness, and helplessness.
	Angry Child	The person experiences intense anger and even rage and feels frustrated and impatient when their needs go unmet.
	Enraged Child	The person experiences excessive anger, leading to out-of-control outbursts of aggression in which he or she may hurt others or destroy objects.
	Impulsive Child	A person acts on impulse or desires. He does not regard the consequences of his/her behavior and has difficulties deferring gratification.
	Undisciplined Child	The individual cannot push himself/herself to complete routine, repetitive tasks as he/she quickly becomes frustrated and gives up.
Dysfunctional Coping Modes	Compliant Surrender	The individual is passive and submissive, requires reassurance and guarantees, and diminishes his/her value because of fear of conflict or rejection.
	Detached Protector	The person escapes the mental pain of unsatisfied needs by turning off all emotions, breaking ties with others, and rejecting anyone’s help. He/she behaves like a robot.
	Detached Self-Soother	The person avoids experiencing emotions by engaging in activities that soothe, stimulate, or distract him or her (such as workaholism, gambling, extreme sports, casual sex, or drug use).
	Self-Aggrandizer	The individual is inclined toward competition and power, behaves pretentiously, downplays, and uses others to achieve what he/she wants. He shows superiority and demands special treatment.
	Bully and Attack	The individual uses threats, bullying, and aggression to achieve what he/she wants or to protect himself/herself from perceived harm.
Dysfunctional Parent Modes	Punitive Parent	It is the internalized voice of significant others who criticize or punish the individual. It results in self-hate, self-denial, self-harm, suicidal fantasies, and self-destructive behavior.
	Demanding Parent	It is a voice that pressures and pushes the individual to meet excessive standards. It expects perfectionism, maintaining order, tidiness, pursuing high status, high productivity, and not wasting time.
Healthy Modes	Healthy Adult	He/she performs functions specific to adults, such as working, raising children, and taking responsibility. He/she also undertakes activities that are a pleasure, such as sex, pursues intellectual, aesthetic, and cultural interests, takes care of his/her health, and plays sports.
	Happy Child	The individual experiences inner peace because his/her basic emotional needs are satisfied. He/she feels loved, fulfilled, competent, secure, praised, valuable, understood, resilient, optimistic, and spontaneous. He/she feels connected and cared for by others. Meanwhile, he/she has a sense of autonomy and control.

**Table 3:** Characteristics of the sample in study.

Demographic Characteristics	Subgroup I	Subgroup II	Subgroup III	Whole Sample
N	39	36	51	126
Gender, N (%):				
Female	22 (56.4%)	26 (72.2%)	41(80.4%)	89 (70.6%)
Male	17 (43.6%)	10 (27.8%)	10 (19.6%)	37 (29.4%)
Age; mean (SD)	26.08 (10.18)	34.00 (13.32)	31.76 (9.17)	30.64 (11.19)
Education; N (%)				
higher	6 (15.4%)	18 (50.0%)	35 (68.6%)	59 (46.8%)
secondary	16 (41.0%)	12 (33.3%)	14 (27.5%)	42 (33.3%)
basic vocational	1 (2.6%)	2 (5.6%)	0 (0.0%)	3 (2.4%)
elementary	16 (41.0%)	4 (11.1%)	2 (3.9%)	22 (17.5%)
Place of residence				
village	7 (17.9%)	9 (25.0%)	12 (23.5%)	28 (22.2%)
city under 100,000 inhabitants	8 (20.5%)	4 (11.1%)	11 (21.6%)	23 (18.3%)
city over 100,000 inhabitants	24 (75.0%)	23 (63.9%)	28 (54.9%)	75 (59.5%)
Marital status				
married	5 (12.8%)	10 (27.8%)	24 (47.1%)	39 (31.0%)
in an informal relationship	11 (28.2%)	9 (25.0%)	18 (35.3%)	38 (30.2%)
single	23 (59.0%)	17 (47.2%)	9 (17.6%)	49 (38.9%)
Employment				
employed/student	18 (46.2%)	28 (77.8%)	49 (96.1%)	95 (75.4%)
retired	1 (2.6%)	0 (0.0%)	0 (0.0%)	1 (0.8%)
unemployed	20 (51.3%)	8 (22.2%)	2 (3.9%)	30 (23.8%)

**Table 4:** Differences between the mean severity of individual symptoms for the first and second measurements in each of the distinguished groups (N = 94).

SCL-90 Subscales/Crisis vs. Stability		Gr. I	Gr. II	Gr. III	Crisis vs. Stability		
					Gr. I	Gr. II	Gr. III
		m (SD)	m (SD)	m (SD)	t	t	t
Somatization	c	1.6 (0.8)	1.4 (1.0)	0.8 (0.8)	0.609 *	0.311 *	−0.096
	s	1.0 (0.7) ↓	1.0 (0.9) ↓	0.8 (0.8)			
Obsessive–compulsive	c	2.3 (0.9)	2.3 (0.7)	1.1 (0.8)	0.491 *	0.427 *	−0.062
	s	1.8 (1.0) ↓	1.8 (0.7) ↓	1.2 (1.0)			
Interpersonal severity	c	2.4 (1.0)	2.0 (0.9)	1.0 (0.8)	0.559 *	0.274 *	−0.069
	s	1.8 (1.1) ↓	1.6 (0.8) ↓	1.1 (0.9)			
Depression	c	2.9 (0.8)	2.4 (0.8)	1.1 (0.9)	1.060 *	0.569 *	0.024
	s	1.8 (0.9) ↓	1.8 (0.8) ↓	1.1 (0.9)			
Anxiety	c	2.4 (1.0)	2.0 (0.9)	0.9 (0.8)	0.703 *	0.530 *	0.031
	s	1.6 (1.1) ↓	1.5 (1.0) ↓	0.9 (0.9)			
Anger and hostility	c	1.5 (1.2)	1.0 (0.8)	0.6 (0.7)	0.510 *	0.328 *	−0.068
	s	0.9 (0.9) ↓	0.7 (0.5) ↓	0.7 (0.7)			
Phobia	c	1.7 (1.1)	1.3 (0.9)	0.4 (0.5)	0.411 *	0.324 *	−0.036
	s	1.2 (1.1) ↓	1.0 (0.8) ↓	0.4 (0.6)			
Paranoid ideation	c	2.0 (1.1)	1.7 (0.9)	0.8 (0.8)	0.536 *	0.361 *	−0.224 *
	s	1.4 (1.1) ↓	1.3 (0.9) ↓	1.0 (0.9) ↑			
Psychoticism	c	1.7 (0.7)	1.4 (0.6)	0.6 (0.7)	0.625 *	0.350 *	−0.091
	s	1.0 (0.7) ↓	1.0 (0.6) ↓	0.6 (0.8)			

Note: m(SD) = mean (standard deviation); c = measurement in crisis; s = measurement in stability; ↓ = the level of severity of an SCL-90 subscale was statistically significantly lower than in crisis; ↑ = the level of severity of an SCL-90 subscale was statistically significantly higher than in crisis; * *p* < 0.05.

**Table 5:** Interaction effects for EMSs in distinguished groups over time (crisis vs. stability * I, II, III).

Schema Domains and EMSs	SS	df	MS	F	η_p_^2^
Disconnection/rejection					
Abandonment/instability	5.799	2	2.900	7.317 ***	0.139
Mistrust/abuse	3.493	2	1.747	4.536 *	0.091
Emotional deprivation	0.705	2	0.353	0.823	0.018
· I, II, III	29.308	2	14.654	5.697 **	0.111
· crisis vs. stability	2.266	1	2.266	5.290 *	0.055
Defectiveness/shame	5.997	2	2.999	8.202 ***	0.153
Social isolation/alienation	4.085	2	2.043	7.977 ***	0.149
Impaired autonomy/performance					
Dependence/incompetence	3.349	2	1.674	7.364 ***	0.139
Vulnerability to harm and illness	2.778	2	1.389	4.621 *	0.092
Enmeshment/undeveloped self	0.996	2	0.498	1.858	0.039
· I, II, III	19.105	2	9.553	5.280 **	0.104
· crisis vs. stability	0.347	1	0.347	1.293	0.014
Failure	2.273	2	1.137	2.503	0.052
· I, II, III	76.084	2	38.042	10.676 ***	0.190
· crisis vs. stability	3.300	1	3.300	7.267 **	0.074
Impaired limits					
Entitlement/grandiosity	0.251	2	0.126	0.566	0.012
· I, II, III	0.122	2	0.061	0.046	0.001
· crisis vs. stability	0.008	1	0.008	0.035	0.000
Insufficient self-control/self-discipline	0.879	2	0.440	1.424	0.030
· I, II, III	19.433	2	9.717	3.946 *	0.080
· crisis vs. stability	3.371	1	3.371	10.914 ***	0.107
Other-directedness					
Subjugation	1.266	2	0.633	2.393	0.050
· I, II, III	27.851	2	13.925	7.675 ***	0.144
· crisis vs. stability	2.270	1	2.270	8.579 **	0.086
Self-sacrifice	1.764	2	0.882	3.400 *	0.070
Approval seeking/recognition seeking	0.128	2	0.064	0.216	0.005
· I, II, III	3.186	2	1.593	0.640	0.014
· crisis vs. stability	1.989	1	1.989	6.747 *	0.069
Hyper vigilance/inhibition					
Negativity/Pessimism	2.270	2	1.135	2.936	0.061
· I, II, III	50.138	2	25.069	8.395 ***	0.156
· crisis vs. stability	3.605	1	3.605	9.327 **	0.093
Emotional inhibition	2.158	2	1.079	5.366 **	0.105
Unrelenting standards/hypercriticalness	0.738	2	0.369	1.601	0.034
· I, II, III	0.087	2	0.043	0.021	0.000
· crisis vs. stability	2.129	1	2.129	9.233 **	0.092
Punitiveness	4.710	2	2.355	6.081 **	0.118

Note: * *p* < 0.05; ** *p* < 0.01; *** *p* < 0.001.

**Table 6:** Interaction effects for schema mode in distinguished groups over time (crisis vs. stability * I, II, III).

Schema Modes	SS	df	MS	F	η_p_^2^
Child modes					
Vulnerable Child	8.223	2	4.112	10.451 ***	0.187
Angry Child	1.411	2	0.705	3.714 *	0.075
Enraged Child	1.065	2	0.532	2.479	0.052
· I, II, III	18.854	2	9.427	7.612 ***	0.143
· crisis vs. stability	2.973	1	2.973	13.844 ***	0.132
Impulsive Child	2.202	2	1.101	4.349 *	0.087
Undisciplined Child	2.062	2	1.031	2.847	0.059
· I, II, III	9.701	2	4.850	2.151	0.045
· crisis vs. stability	5.292	1	5.292	14.615 ***	0.138
Dysfunctional coping modes					
Compliant Surrender	2.758	2	1.379	5.552 **	0.109
Detached Protector	7.706	2	3.853	11.229 ***	0.198
Detached Self-Soother	0.269	2	0.135	0.439	0.010
· I, II, III	11.846	2	5.923	2.897	0.060
· crisis vs. stability	4.560	1	4.560	14.866 ***	0.140
Self-Aggrandizer	0.187	2	0.094	0.737	0.016
· I, II, III	2.856	2	1.428	1.106	0.024
· crisis vs. stability	0.120	1	0.120	0.947	0.010
Bully and Attack	0.660	2	0.330	1.680	0.036
· I, II, III	7.799	2	3.899	4.067 *	0.082
· crisis vs. stability	1.504	1	1.504	7.659 **	0.078
Dysfunctional parent modes					
Punitive Parent	4.926	2	2.463	8.384 ***	0.156
Demanding Parent	0.329	2	0.164	0.686	0.015
· I, II, III	0.463	2	0.231	0.173	0.004
· crisis vs. stability	2.271	1	2.271	9.470 **	0.094
Healthy modes					
Healthy Adult	1.754	2	0.877	3.431 *	0.070
Happy Child	2.358	2	1.179	3.700 *	0.075

Note: * *p* < 0.05; ** *p* < 0.01; *** *p* < 0.001.

**Table 7:** Detailed intergroup comparisons of means and effect sizes for EMSs.

EMSs/Crisis vs. Stability		Gr. I	Gr. II	Gr. III	Gr. I vs. Gr. II		Gr. I vs. Gr. III	
		m (SD)	m (SD)	m (SD)	t	r	t	r
Disconnection/rejection								
Abandonment/instability	c	4.8 (1.1)	3.5 (1.2) ↓	2.8 (1.3) ↓	4.473 ***	0.425	7.660 ***	0.626
	s	4.1 (1.2)	3.6 (1.2)	2.8 (1.3) ↓	1.721	0.178	4.437 ***	0.422
Mistrust/abuse	c	4.2 (1.5)	3.2 (1.2) ↓	2.3 (1.1) ↓	3.484 ***	0.343	6.399 ***	0.557
	s	3.5 (1.5)	2.9 (1.2)	2.1 (1.1) ↓	1.810	0.186	4.452 ***	0.423
Emotional deprivation	c	3.2 (1.4)	3.0 (1.3)	2.1 (1.2) ↓	0.985	0.103	3.503 ***	0.345
	s	2.9 (1.3)	2.5 (1.3)	2.1 (1.1) ↓	1.142	0.119	2.674 **	0.270
Defectiveness/shame	c	4.1 (1.4)	2.9 (1.1) ↓	1.7 (1.0) ↓	3.795 ***	0.370	7.715 ***	0.629
	s	3.1 (1.6)	2.6 (1.2)	1.7 (1.0) ↓	1.536	0.159	4.458 ***	0.423
Social isolation/alienation	c	4.4 (1.4)	3.7 (1.3) ↓	2.3 (1.2) ↓	2.918 **	0.292	7.042 ***	0.594
	s	3.9 (1.5)	3.5 (1.5)	2.4 (1.1) ↓	1.295	0.134	4.401 ***	0.419
Impaired autonomy/performance								
Dependence/ incompetence	c	3.6 (1.4)	3.1 (1.1)	1.8 (0.8) ↓	0.765	0.080	5.384 ***	0.492
	s	2.7 (1.3)	3.0 (1.0)	1.8 (1.0) ↓	−1.118	0.116	3.314 ***	0.328
Vulnerability to harm and illness	c	3.7 (1.4)	3.3 (1.2)	2.3 (1.2) ↓	1.107	0.115	4.409 ***	0.420
	s	3.0 (1.3)	3.1 (1.1)	2.2 (1.3) ↓	−0.238	0.025	2.556 *	0.259
Enmeshment/ undeveloped self	c	2.5 (1.1)	2.6 (0.9)	1.7 (0.7) ↓	−0.342	0.036	3.007 **	0.301
	s	2.1 (0.9)	2.5 (1.2)	1.8 (1.2)	−1.278	0.133	1.372	0.142
Failure	c	4.3 (1.4)	3.7 (1.3)	2.3 (1.3) ↓	1.024	0.107	5.012 ***	0.465
	s	3.5 (1.7)	3.4 (1.3)	2.3 (1.4) ↓	0.125	0.013	3.142 **	0.313
Impaired limits								
Entitlement/grandiosity	c	2.8 (1.0)	2.8 (2.8)	2.7 (0.9)	0.692	0.072	0.346	0.036
	s	2.7 (0.9)	2.7 (0.8)	2.7 (1.0)	−0.121	0.013	−0.028	0.003
Insufficient self-control/ self-discipline	c	3.9 (1.4)	3.7 (1.1)	2.8 (1.2) ↓	0.468	0.049	2.891 **	0.290
	s	3.3 (1.1)	3.4 (1.1)	2.8 (1.2)	−0.118	0.012	1.867	0.192
Other-directedness								
Subjugation	c	3.5 (1.2)	3.0 (1.0)	2.3 (1.1) ↓	1.459	0.151	4.425 ***	0.421
	s	2.9 (1.2)	2.7 (0.9)	2.2 (1.0) ↓	0.862	0.090	2.782 **	0.280
Self-sacrifice	c	4.0 (1.3)	3.1 (1.0) ↓	3.3 (1.0) ↓	3.333 ***	0.330	2.926 **	0.293
	s	3.7 (1.3)	3.0 (1.1) ↓	3.3 (1.0)	2.204 *	0.225	1.213	0.126
Approval seeking/ recognition seeking	c	3.7 (1.2)	3.9 (1.1)	3.3 (1.2)	−0.281	0.029	0.889	0.093
	s	3.5 (1.2)	3.4 (1.0)	3.2 (1.3)	0.114	0.012	0.891	0.093
Hyper vigilance/inhibition								
Negativity/pessimism	c	4.4 (1.2)	3.9 (1.3)	2.9 (1.4) ↓	1.346	0.140	4.643 ***	0.438
	s	3.8 (1.3)	3.5 (1.1)	2.8 (1.5) ↓	0.603	0.063	2.847 **	0.286
Emotional inhibition	c	3.6 (1.3)	3.5 (1.2)	2.6 (1.1) ↓	0.743	0.078	3.368 ***	0.333
	s	3.0 (1.1)	3.1 (1.3)	2.5 (1.1)	−0.357	0.037	1.701	0.176
Unrelenting standards/ hypercriticalness	c	3.5 (1.1)	3.8 (0.9)	3.5 (1.1)	−0.421	0.044	0.357	0.037
	s	3.5 (1.0)	3.4 (1.0)	3.5 (1.2)	0.248	0.026	−0.113	0.012
Punitiveness	c	3.7 (1.5)	3.3 (1.1)	2.5 (1.2) ↓	1.336	0.139	4.339 ***	0.414
	s	3.1 (1.4)	2.8 (1.0)	2.4 (1.1) ↓	0.891	0.093	2.168 *	0.222

Note: m(SD) = mean (standard deviation); c = measurement in crisis; s = measurement in stability; ↓ = the level of severity of a specific EMS was statistically significantly lower than that in Group I; * *p* < 0.05; ** *p* < 0.01; *** *p* < 0.001.

**Table 8:** Detailed intergroup comparisons of mean and effect sizes for schema modes.

Schema Modes/ Crisis vs. Stability		Gr. I	Gr. II	Gr. III	Gr.I vs. Gr.II		Gr.I vs. Gr.III	
		m (SD)	m (SD)	m (SD)	t	r	t	r
Child modes								
Vulnerable Child	c	4.8 (0.9)	4.0 (1.1) ↓	2.5 (1.1) ↓	3.619 ***	0.355	8.218 ***	0.653
	s	3.4 (1.1)	3.2 (1.1)	2.3 (1.1) ↓	0.854	0.089	4.075 ***	0.393
Angry Child	c	3.8 (0.9)	3.2 (0.8) ↓	2.9 (0.9) ↓	2.928 **	0.293	4.393 ***	0.418
	s	3.3 (1.0)	3.0 (0.9)	2.6 (0.7) ↓	1.125	0.117	2.808 **	0.282
Enraged Child	c	2.5 (1.3)	1.9 (0.8) ↓	1.6 (0.6) ↓	2.542 *	0.257	3.958 ***	0.383
	s	2.0 (1.0)	1.6 (0.5) ↓	1.5 (0.6) ↓	2.186 *	0.223	3.037 **	0.303
Impulsive Child	c	3.5 (1.2)	2.8 (1.0) ↓	2.2 (0.9) ↓	2.902 **	0.291	5.330 ***	0.488
	s	2.7 (1.0)	2.3 (0.8)	1.9 (0.8) ↓	1.719	0.177	3.636 ***	0.356
Undisciplined Child	c	4.0 (1.2)	3.8 (1.0)	3.0 (1.2) ↓	0.579	0.061	2.615 *	0.264
	s	3.3 (1.3)	3.4 (0.9)	3.1 (1.2)	−0.408	0.043	0.826	0.086
Dysfunctional coping modes								
Compliant Surrender	c	3.7 (1.1)	3.1 (0.8) ↓	2.8 (1.0) ↓	2.044 *	0.210	3.385 **	0.334
	s	2.9 (0.9)	2.9 (0.8)	2.7 (0.9)	0.201	0.021	0.978	0.102
Detached Protector	c	3.7 (0.9)	3.1 (1.2) ↓	2.1 (1.1) ↓	2.585 *	0.262	5.761 ***	0.517
	s	2.7 (0.9)	2.6 (1.2)	2.2 (1.2)	0.353	0.037	1.868	0.192
Detached Self-Soother	c	3.9 (1.0)	3.4 (1.2)	3.1 (1.1) ↓	1.679	0.173	2.417 *	0.246
	s	3.4 (1.0)	3.1 (1.2)	2.8 (1.2) ↓	0.930	0.097	2.065 *	0.212
Self-Aggrandizer	c	2.6 (0.8)	2.8 (0.9)	2.9 (0.9)	−0.214	0.022	−1.449	0.150
	s	2.7 (0.8)	2.6 (0.8)	2.9 (0.8)	0.423	0.044	−0.908	0.095
Bully and Attack	c	2.3 (0.8)	2.2 (0.9)	1.8 (0.7) ↓	0.904	0.094	3.095 **	0.309
	s	2.0 (0.9)	2.0 (0.7)	1.7 (0.6)	0.071	0.007	1.790	0.184
Dysfunctional parent modes								
Punitive Parent	c	3.8 (1.2)	3.0 (0.8) ↓	2.0 (0.9) ↓	2.698 **	0.272	6.477 ***	0.562
	s	2.7 (1.0)	2.4 (0.9)	1.8 (0.8) ↓	1.211	0.126	3.723 ***	0.364
Demanding Parent	c	3.8 (0.8)	3.7 (0.8)	3.7 (0.9)	0.586	0.061	0.607	0.064
	s	3.5 (0.8)	3.4 (0.9)	3.6 (1.0)	0.482	0.050	−0.233	0.024
Healthy modes								
Healthy Adult	c	3.0 (0.8)	3.3 (0.8)	4.4 (0.8) ↑	−1.013	0.106	−6.801 ***	0.581
	s	3.6 (0.8)	3.7 (0.8)	4.5 (0.7) ↑	−0.215	0.023	−4.589 ***	0.434
Happy Child	c	2.1 (0.7)	2.5 (0.9)	3.9 (1.1) ↑	−1.844	0.190	−7.496 ***	0.618
	s	2.9 (1.0)	3.0 (1.2)	4.0 (0.9) ↑	−0.079	0.008	−4.397 ***	0.419

Note: m(SD) = mean (standard deviation); c = measurement in crisis; s = measurement in stability; ↓ = the level of severity of a specific schema mode was statistically significantly lower than in Group I; ↑ = the level of severity of a specific schema mode was statistically significantly higher than in Group I; * *p* < 0.05; ** *p* < 0.01; *** *p* < 0.001.

**Table 9:** Detailed intragroup comparisons of mean and effect sizes for EMSs.

EMSs/Crisis vs. Stability		Gr. I	Gr. II	Gr. III	Crisis vs. Stability					
					Gr. I		Gr. II		Gr. III	
		m (SD)	m (SD)	m (SD)	t	r	t	r	t	r
Disconnection/rejection										
Abandonment/instability	c	4.8 (1.1)	3.5 (1.2)	2.8 (1.3)	3.614 ***	0.354	−0.957	0.100	−1.191	0.124
	s	4.1 (1.2) ↓	3.6 (1.2)	2.8 (1.3)						
Mistrust/abuse	c	4.2 (1.5)	3.2 (1.2)	2.3 (1.1)	3.868 ***	0.376	0.401	0.042	−0.040	0.004
	s	3.5 (1.5) ↓	2.9 (1.2)	2.1 (1.1)						
Emotional deprivation	c	3.2 (1.4)	3.0 (1.3)	2.1 (1.2)	1.681	0.174	1.976	0.203	0.306	0.032
	s	2.9 (1.3)	2.5 (1.3)	2.1 (1.1)						
Defectiveness/shame	c	4.1 (1.4)	2.9 (1.1)	1.7 (1.0)	5.954 ***	0.529	1.730	0.178	0.455	0.048
	s	3.1 (1.6) ↓	2.6 (1.2)	1.7 (1.0)						
Social isolation/alienation	c	4.4 (1.4)	3.7 (1.3)	2.3 (1.2)	4.595 ***	0.434	0.796	0.083	−0.939	0.098
	s	3.9 (1.5) ↓	3.5 (1.5)	2.4 (1.1)						
Impaired autonomy/performance										
Dependence/ incompetence	c	3.6 (1.4)	3.1 (1.1)	1.8 (0.8)	4.666 ***	0.439	0.259	0.027	−0.315	0.033
	s	2.7 (1.3) ↓	3.0 (1.0)	1.8 (1.0)						
Vulnerability to harm and illness	c	3.7 (1.4)	3.3 (1.2)	2.3 (1.2)	3.922 ***	0.380	0.824	0.086	−0.228	0.024
	s	3.0 (1.3) ↓	3.1 (1.1)	2.2 (1.3)						
Enmeshment/ undeveloped self	c	2.5 (1.1)	2.6 (0.9)	1.7 (0.7)	2.221 *	0.227	0.105	0.011	−0.338	0.035
	s	2.1 (0.9) ↓	2.5 (1.2)	1.8 (1.2)						
Failure	c	4.3 (1.4)	3.7 (1.3)	2.3 (1.3)	3.228 **	0.320	1.374	0.143	0.074	0.008
	s	3.5 (1.7) ↓	3.4 (1.3)	2.3 (1.4)						
Impaired limits										
Entitlement/grandiosity	c	2.8 (1.0)	2.8 (2.8)	2.7 (0.9)	0.849	0.089	−0.658	0.069	0.159	0.017
	s	2.7 (0.9)	2.7 (0.8)	2.7 (1.0)						
Insufficient self-control/ self-discipline	c	3.9 (1.4)	3.7 (1.1)	2.8 (1.2)	3.149 **	0.313	1.812	0.187	0.765	0.080
	s	3.3 (1.1) ↓	3.4 (1.1)	2.8 (1.2)						
Other-directedness										
Subjugation	c	3.5 (1.2)	3.0 (1.0)	2.3 (1.1)	3.111 **	0.310	1.907	0.196	0.049	0.005
	s	2.9 (1.2) ↓	2.7 (0.9)	2.2 (1.0)						
Self-sacrifice	c	4.0 (1.3)	3.1 (1.0)	3.3 (1.0)	2.553 *	0.258	0.167	0.018	−1080	0.112
	s	3.7 (1.3) ↓	3.0 (1.1)	3.3 (1.0)						
Approval seeking/ recognition seeking	c	3.7 (1.2)	3.9 (1.1)	3.3 (1.2)	1.197	0.125	1.997 *	0.205	1.289	0.134
	s	3.5 (1.2)	3.4 (1.0) ↓	3.2 (1.3)						
Hyper vigilance/inhibition										
Negativity/pessimism	c	4.4 (1.2)	3.9 (1.3)	2.9 (1.4)	3.418 ***	0.337	1.869	0.192	0.101	0.011
	s	3.8 (1.3) ↓	3.5 (1.1)	2.8 (1.5)						
Emotional inhibition	c	3.6 (1.3)	3.5 (1.2)	2.6 (1.1)	6.188 ***	0.544	3.134 **	0.312	1.617	0.167
	s	3.0 (1.1) ↓	3.1 (1.3) ↓	2.5 (1.1)						
Unrelenting standards/ hypercriticalness	c	3.5 (1.1)	3.8 (0.9)	3.5 (1.1)	1.614	0.167	3.035 **	0.303	0573	0.060
	s	3.5 (1.0)	3.4 (1.0) ↓	3.5 (1.2)						
Punitiveness	c	3.7 (1.5)	3.3 (1.1)	2.5 (1.2)	3.857 ***	0.375	2.738 **	0.276	−0.844	0.088
	s	3.1 (1.4) ↓	2.8 (1.0) ↓	2.4 (1.1)						

Note: m(SD) = mean (standard deviation); c = measurement in crisis; s = measurement in stability; ↓ = the level of severity of a specific EMS was statistically significantly lower than obtained in crisis; * *p* < 0.05; ** *p* < 0.01; *** *p* < 0.001.

**Table 10:** Detailed intragroup comparisons of mean and effect sizes for schema modes.

Schema Modes/Crisis vs. Stability		Gr. I	Gr. II	Gr. III	Crisis vs. Stability					
					Gr. I		Gr. II		Gr. III	
		m (SD)	m (SD)	m (SD)	t	r	t	r	t	r
Child modes										
Vulnerable Child	c	4.8 (0.9)	4.0 (1.1)	2.5 (1.1)	8.250 ***	0.654	3.684 ***	0.360	1.953	0.201
	s	3.4 (1.1) ↓	3.2 (1.1) ↓	2.3 (1.1)						
Angry Child	c	3.8 (0.9)	3.2 (0.8)	2.9 (0.9)	4.761 ***	0.447	1.126	0.117	1.692	0.175
	s	3.3 (1.0) ↓	3.0 (0.9)	2.6 (0.7)						
Enraged Child	c	2.5 (1.3)	1.9 (0.8)	1.6 (0.6)	3.776 ***	0.368	2.043 *	0.209	0.629	0.066
	s	2.0 (1.0) ↓	1.6 (0.5) ↓	1.5 (0.6)						
Impulsive Child	c	3.5 (1.2)	2.8 (1.0)	2.2 (0.9)	5.217 ***	0.480	2.405 *	0.244	1.118	0.116
	s	2.7 (1.0) ↓	2.3 (0.8) ↓	1.9 (0.8)						
Undisciplined Child	c	4.0 (1.2)	3.8 (1.0)	3.0 (1.2)	3.989 ***	0.386	2.017 *	0.207	0.623	0.065
	s	3.3 (1.3) ↓	3.4 (0.9) ↓	3.1 (1.2)						
Dysfunctional coping modes										
Compliant Surrender	c	3.7 (1.1)	3.1 (0.8)	2.8 (1.0)	5.518 ***	0.501	1.998 *	0.205	1.003	0.105
	s	2.9 (0.9) ↓	2.9 (0.8) ↓	2.7 (0.9)						
Detached Protector	c	3.7 (0.9)	3.1 (1.2)	2.1 (1.1)	6.426 ***	0.559	2.375 *	0.242	−0.237	0.025
	s	2.7 (0.9) ↓	2.6 (1.2) ↓	2.2 (1.2)						
Detached Self-Soother	c	3.9 (1.0)	3.4 (1.2)	3.1 (1.1)	2.821 **	0.284	1.457	0.151	2.426 *	0.246
	s	3.4 (1.0) ↓	3.1 (1.2)	2.8 (1.2) ↓						
Self-Aggrandizer	c	2.6 (0.8)	2.8 (0.9)	2.9 (0.9)	−0.429	0.045	1.046	0.109	1.052	0.110
	s	2.7 (0.8)	2.6 (0.8)	2.9 (0.8)						
Bully and Attack	c	2.3 (0.8)	2.2 (0.9)	1.8 (0.7)	2.982 **	0.298	1.415	0.147	0.403	0.042
	s	2.0 (0.9) ↓	2.0 (0.7)	1.7 (0.6)						
Dysfunctional parent modes										
Punitive Parent	c	3.8 (1.2)	3.0 (0.8)	2.0 (0.9)	7.265 ***	0.606	4.097 ***	0.395	1.476	0.153
	s	2.7 (1.0) ↓	2.4 (0.9) ↓	1.8 (0.8)						
Demanding Parent	c	3.8 (0.8)	3.7 (0.8)	3.7 (0.9)	2.333 *	0.238	2.146 *	0.220	0.839	0.088
	s	3.5 (0.8) ↓	3.4 (0.9) ↓	3.6 (1.0)						
Healthy modes										
Healthy Adult	c	3.0 (0.8)	3.3 (0.8)	4.4 (0.8)	−4.451 ***	0.423	−3.064 **	0.306	−0.797	0.083
	s	3.6 (0.8) ↑	3.7 (0.8) ↑	4.5 (0.7)						
Happy Child	c	2.1 (0.7)	2.5 (0.9)	3.9 (1.1)	−5.592 ***	0.506	−2.745 **	0.276	−1.882	0.194
	s	2.9 (1.0) ↑	3.0 (1.2) ↑	4.0 (0.9)						

Note: m(SD) = mean (standard deviation); c = measurement in crisis; s = measurement in stability; ↓ = the level of severity of a specific schema mode was statistically significantly lower than in crisis; ↑ = the level of severity of a specific schema mode was statistically significantly higher than in crisis; * *p* < 0.05; ** *p* < 0.01; *** *p* < 0.001.

## Data Availability

The data presented in this study are available on request from the corresponding author. The data are not publicly available due to privacy issues.
